# ENGAGING COMMUNITY HOSPICE PROVIDERS TO SUPPORT VETERANS AND THEIR FAMILIES AT END-OF-LIFE

**DOI:** 10.1093/geroni/igae098.2026

**Published:** 2024-12-31

**Authors:** Deanna Dragan, Lauren Hagemann, Cynthia Jarmul

**Affiliations:** Salem VAMC, Salem, Virginia, United States; Salem VA Healthcare System, Salem, Virginia, United States; Salem VA Medical Center, Salem, Virginia, United States

## Abstract

The VHA’s Empowering Community Hospices Initiative was developed to help strengthen the VHA’s coordination of care practices with non-VA providers. It is imperative that community hospice providers (CHP) be privy to the unique experiences of Veterans as their needs may be significantly different than those of a non-veteran at end-of-life (EOL) like specialized care and benefits for conditions such as PTSD, Moral injury and suicidality. For this project we completed a formal program evaluation study in collaboration with CHPs to explore the potential barriers and facilitators for creating and sustaining partnerships. Data collection included semi-structured interviews with stakeholders (n = 18) across multiple disciplines (e.g., Social Workers, Psychologists, Physicians, etc.) at the National, VISN, and local levels. Also, we conducted outreach efforts to local CHPs (n = 8), completed semi-structured interviews (n = 4), and coordinated face-to-face site visits (n = 4) for the VHA team to meet with the teams at each agency. These interviews revealed themes related to perceived barriers to and facilitators for achieving the goals of the initiative. Themes identified included: point of contact, timeline of hospice care, differences per agency, and need for resources to support Veterans’ families. CHPs collaborated with VHA to develop feasible partnerships with plans for sustainability using a consultation model of care. It is anticipated that the demand for EOL services may soon exceed the supply of care that VA facilities can provide; thus, these efforts may improve the access to services for Veterans in community hospice care settings.

